# Impact of Bock Beer Marination on the Physicochemical, Functional and Technological Properties of Minas Artisanal Authorial Cheese

**DOI:** 10.17113/ftb.64.02.26.9417

**Published:** 2026-06-15

**Authors:** Guilherme Cardoso de Almeida, Larissa Karla de Jesus, Giselle Pereira Cardoso, Caique Menezes de Abreu, Raquel Guidetti Vendruscolo, Larissa de Oliveira Ferreira-Rocha

**Affiliations:** Institute of Science and Technology, Federal University of Jequitinhonha and Mucuri Valleys, MGT-367 Highway, Km 583, n. 5000, Diamantina, MG 39100-000, Brazil

**Keywords:** artisanal cheese, bock beer marination, fatty acid profile, phenolic compounds, proteolysis index

## Abstract

**Research background:**

Various technologies can enhance the quality and sensory characteristics of foods. Marination, for example, aims to add or intensify flavor, aroma and color, improve texture, promote biopreservation, and differentiate and add value to products. The use of alcoholic beverages for cheese marination has recently attracted interest. Although the effects depend on the type of beverage, immersion time, and cheese variety, all studies report significant physicochemical and sensory changes. In this context, the present study aims to provide artisanal producers with an innovative and scientifically grounded method to add value and expand their market opportunities.

**Experimental approach:**

This study explores the effects of bock beer marination on the technological and functional quality of Minas artisanal cheese from the Diamantina region, ripened for 22 days. Different marination times were evaluated: 0 (C), 2 (T2), 4 (T4), and 6 (T6) days, focusing on physicochemical characteristics, proteolysis index, phenolic compound content, fatty acid profile, color and texture.

**Results and conclusions:**

Beer marination significantly altered the composition and technological properties of the cheese samples. Moisture increased in T4 and stabilized in T6, while ash mass fraction progressively decreased, and variations were observed in lipids and proteins. In contrast, carbohydrates, fatty acids, pH, and water activity remained stable. Phenolic compounds showed a non-significant increasing trend, along with intensified proteolysis, particularly in T4 and T6. The rind exhibited a more intense coloration than the interior, and texture varied over time, with greater firmness in T4 and softening in T6. Beer marination demonstrated potential as an innovative strategy for differentiating Minas artisanal cheese.

**Novelty and scientific contribution:**

This study presents a pioneering strategy to enhance the production of Minas artisanal cheese, demonstrating that marination can be a viable alternative to add value and diversify artisanal cheese samples without compromising their authenticity and traditional identity.

## INTRODUCTION

Various types of artisanal cheese are produced worldwide, playing a significant role in sustainable agriculture and strengthening local economies (*1*). In this context, Brazilian artisanal cheeses hold cultural, economic and gastronomic importance, being unique products that represent the cultural heritage and identity of their regions of origin (*2*, *3*).

Artisanal cheese production is widespread in Brazil, particularly in Minas Gerais (*4*). Currently, ten officially recognized regions produce Minas artisanal cheese: Araxá, Campos das Vertentes, Cerrado, Serra da Canastra, Serra do Salitre, Serro, Triângulo Mineiro, Serras da Ibitipoca, Diamantina, and Entre Serras da Piedade ao Caraça. Other regions in Minas Gerais, such as Alagoa, Mantiqueira de Minas, Serra Geral do Norte de Minas, Vale do Jequitinhonha, and Vale do Suaçuí, are also known for their artisanal cheese-making traditions (*5*).

According to Instituto Mineiro de Agropecuária (IMA) Ordinance No. 2322 (*6*), Minas artisanal cheese produced in the Diamantina region exhibits defined sensory characteristics, including semi-hard consistency, granular texture, yellowish color, slightly pungent flavor, and smooth rind, with a minimum ripening time of 22 days. In addition to its unique sensory attributes, this cheese has been crafted for centuries using techniques passed down through generations, embodying the region's cultural identity. However, investing in innovative techniques that add value to the product is essential to enhance its market competitiveness and achieve broader recognition.

Some well-established technologies, such as ripening, smoking, and marination, can enhance cheese quality and sensory characteristics (*7*). Marination is a processing technique that involves the immersion in or incorporation of seasoned liquid marinades, either raw or cooked (*8*). In the marination process, salts, organic acids, fruits and vegetables, beverages such as soft drinks and wine, enzymes, and vinegar, as well as combinations of these agents, are commonly used (*9*). This technique aims to add or intensify flavor, aroma, and color, improve texture, promote biopreservation, and differentiate and add value to cheeses (*8*, *10*).

Marinating cheese in alcoholic beverages has recently attracted interest. For example, cachaça has been used to marinate queijo coalho (*11*), while wines have been applied to colonial cheese (*12*) and hard cheese (*13*). Although the effects vary according to the beverage, immersion time, and cheese type, all studies observed physicochemical and sensory modifications. Generally, marination influences texture, color, and sensory profile, while contributing to functional attributes such as antioxidant activity and microbiological safety.

Although alcoholic beverages have been explored in cheese marination, the use of beer has not yet been investigated in this context. Previous studies on meat, such as those by Manful *et al.* (*14*), showed that beer-based marinades can improve its sensory quality and oxidative stability. Given these promising findings, beer marination may represent an innovative approach for enhancing artisanal cheese sensory, functional and technological value.

Beer is a fermented beverage made from barley malt, water, hops and yeast, and is one of the most consumed alcoholic beverages worldwide (*15*). In addition to ethanol, beer contains significant amounts of bioactive compounds, including phenolics, bitter acids derived from hops (alpha and iso-alpha acids), soluble proteins, B-complex vitamins, and minerals (*16*–*18*). The composition and characteristics of beer vary according to the raw materials used and specific production methods.

Beyond sensory and technological changes, the presence of bioactive compounds is of great interest due to their potential to enhance the functional value of artisanal cheese through beer-based marination. Dark and bock beer styles stand out because they use more roasted malts, increasing the release of phenolic compounds during brewing (*19*). Moreover, other bioactive molecules derived from the Maillard reaction, such as melanoidins, are also formed (*20*, *21*).

Studies have demonstrated that beer is a relevant source of bioactive compounds, particularly polyphenols and melanoidins, which exert functional effects associated with health promotion. Polyphenols, mainly derived from malt and hops, exhibit high antioxidant capacity, contributing to the neutralization of free radicals and the reduction of the risk of chronic diseases, including cardiovascular disorders, cancer, type II diabetes, and neurodegenerative conditions (*22*, *23*). Among these compounds, xanthohumol and its derivatives, exclusively found in hops, have been extensively investigated due to their anticancer, anti-inflammatory, and hypocholesterolaemia properties. Melanoidins, formed during Maillard reactions in beer processing, not only influence sensory parameters but also display potential antioxidant, antimicrobial, antihypertensive and prebiotic activities (*23*). Recent evidence indicates that moderate beer consumption may provide health benefits, such as improved bone health, promotion of intestinal balance, reduction of cholesterol, and enhancement of vascular function, effects mainly attributed to its non-alcoholic fraction rich in phenolic compounds (*24*).

In this context, although beer-based marination shows potential technological, sensory, and functional benefits when applied to foods, no studies have yet investigated its impact on artisanal cheese. Therefore, the present study aims to evaluate the influence of bock beer marination on the technological and functional quality of Minas artisanal cheese ripened for 22 days, with emphasis on physicochemical characteristics, proteolysis index, phenolic content, fatty acid profile, color and texture.

## MATERIALS AND METHODS

### Cheese production

The cheeses were produced following the official production process for Minas artisanal cheese (*25*), with adaptations that included the addition of a marination step.

**Fig. S1:**
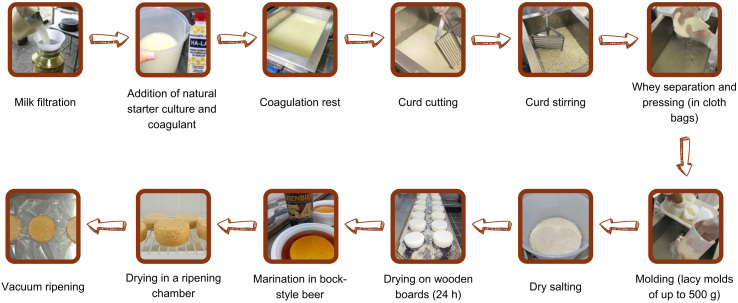
Flowchart of cheese production and marination process

As shown in Fig. S1, the manufacturing process began immediately after milking. Milk from Holstein cows was sourced from the Dairy Cattle Sector of the Federal University of the Jequitinhonha and Mucuri Valleys (UFVJM), JK Campus, Diamantina, Brazil. After filtration, the milk was transferred to production vats, and the natural starter culture (pingo), also known as whey starter and supplied by the Braúnas cheese factory (Diamantina, Brazil), was added. The coagulant agent (HA-LA®, Chr. Hansen, Valinhos, Brazil), containing the enzyme chymosin, was then added. After coagulation for 40 min, the curd was cut and stirred to promote whey expulsion. Previously sanitized stainless-steel sieves were used to collect the curd mass, which was placed in cloth bags and manually pressed to enhance whey drainage. Molds with perforated bottom and a maximum capacity of 500 g were used for molding. After molding, dry salting was carried out: coarse salt (10 g; Pachá®, Contagem, Brazil) was applied to one surface of the cheese, which was then left to rest on the bench for 6 h. The cheese was then turned over, and the procedure was repeated on the opposite surface with an additional 10 g of salt. After salting, the cheese samples were removed from the molds and dried for 24 h on a wooden board (provided by the cheese factory). After drying, the cheese samples were marinated.

For marination, each cheese sample (approx. 250 g) was fully submerged in 150 mL of bock-style beer in sealed polystyrene containers and maintained at (5±1) °C. Three cheese samples were used for each treatment. The beer was selected for its high phenolic content (expressed as gallic acid equivalents, 478 mg/L), determined among beer samples of the same brand but different styles. The selected bock beer (Wienbier®, Leme, Brazil) had an international bitterness unit (IBU) of 15, a European Brewery Convention (EBC) value of 30, an alcohol content of 5.0%, and the following ingredients: water, malt, hops, and stabilizer International Numbering System (INS) 405.

To ensure uniform marination, the cheese samples were turned every 24 h during the immersion periods of 2 (T2), 4 (T4) and 6 days (T6). After marination, the samples were dried in a ripening chamber at (12±1) °C for 72 h, vacuum-packed in polyethylene pouches, and stored in the chamber at the same temperature for an additional 22 days. Control samples (C) underwent the same processing steps, except for beer marination.

Cheese production was carried out in a single batch using the same milk for all treatments, in which all experimental units were manufactured. After the salting and drying steps, the cheese samples were randomly assigned to the treatments for the marination stage, totaling five units per treatment and five units for the control group. Subsequently, three of the five units from each group were randomly selected for analyses. After color and texture analyses, which required intact samples, the cheese samples were grated to promote homogenization and enable the remaining analyses.

#### pH, color, melanoidins, and phenolic compounds of beer

The pH, instrumental color, melanoidins, and phenolic compounds of beer samples were evaluated. Before analysis, the samples were degassed using an ultrasonic bath (SSBUC-20L; SolidSteel, Piracicaba, Brazil).

The pH was measured directly in 20 mL of beer using the electrometric method with a digital pH meter (mPA-210; MS Tecnopon, Piracicaba, Brazil).

Color was assessed by instrumental colorimetry according to the CIELAB system, with D65 illuminant, 10° viewing angle, and SCI mode calibration (specular component included), using a spectrophotometer (CM-5; Konica Minolta, Chiyoda, Japan), as described in the section Color parameters.

Melanoidins were determined according to Tagliazucchi *et al.* (*26*). The preparation involved diluting 1 mL of beer to a final volume of 5 mL with distilled water, and measuring the absorbance at 420 nm using a single-beam UV-Vis spectrophotometer (UV-M5; Bel Photonics, Monza, Italy). The melanoidin content (g/L) was calculated according to the following equation:


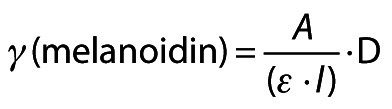
 /1/

where *A* is the absorbance at 420 nm, *ε* is the melanoidin molar absorption coefficient (0.5838) in beer measured at 420 nm (*26*), *l* is the cuvette path length (in cm), and D is the sample dilution.

Total soluble phenolic compounds were quantified using the Folin-Ciocalteu spectrophotometric method (*27*). For this, 200 μL of beer were mixed with 1.9 mL of Folin-Ciocalteu reagent. After 3 min, 1 mL of 20% aqueous sodium carbonate solution and 2 mL of distilled water were added. The mixture was incubated in the dark for 30 min to allow color development, and the absorbance was measured at 750 nm using the same UV-Vis spectrophotometer. The samples were quantified using a gallic acid standard curve (0–600 mg/mL), and results were expressed as mg of gallic acid equivalents per liter of beer (mg/L). All analyses were performed in triplicate.

#### Cheese evaluation methods

All analyses for cheese characterization were performed in triplicate.

### Physicochemical analyses and total soluble phenolic content

Moisture content was determined using 5.0 g of sample by the gravimetric method at an oven (400-4TS/td; EthikTechnology, Vargem Grande Paulista, Brazil) temperature of 105 °C (*28*), and ash content was determined by incinerating 5.0 g of sample in a muffle furnace at 550 °C (*29*). Protein content was determined with a sample of 0.25 g using the Kjeldahl method, multiplying the total nitrogen by 6.38 (*30*). Lipid content was quantified with 1.0 g of sample using the method of Bligh and Dyer (*31*). Total carbohydrates were calculated by difference in the moisture, fat, protein and ash contents of the samples.

The pH of the samples was measured with 10 g of sample and 20 mL of distilled water with the electrometric method and a digital pH meter (mPA-210; MS Tecnopon). Water activity (*a*_w_) was assessed at 25 °C using a water activity meter (4TE Duo; AquaLab, Pullman, WA, USA).

Total soluble phenolic content (TSPC) was determined using the Folin–Ciocalteu method with modifications as described by El Hatmi *et al*. (*32*). A mass of 2 g of wet cheese was weighed into a Falcon tube, and 3 mL of analytical-grade methanol (Isofar, Duque de Caxias, Brazil) was added as the extracting solution. The mixture was vortexed (NA 3600; Norte Científica, Araraquara, Brazil) for 2 min and subjected to an ultrasonic bath (CBU/100/3LDG; Planatec, São Paulo, Brazil) at 40 kHz and 100 W for 5 min. After sonication, solid–liquid separation was optimized by centrifugation (SL-5GR; Spinlab, Ribeirão Preto, Brazil) at 7000×*g* and 4 °C for 10 min. The extraction was exhaustive and repeated in three cycles; the final supernatant was adjusted to a total volume of 10 mL.

For the colorimetric reaction, 100 μL of the extract containing TSPC were mixed with 250 μL of 0.2 M Folin–Ciocalteu reagent (Êxodo Científica, Sumaré, Brazil), 3 mL of distilled water, and 1 mL of 15% aqueous sodium carbonate solution. The mixture was incubated in the dark for 30 min for color development. Absorbance was read at 750 nm using a single-beam UV-Vis spectrophotometer (UV-M5; Bel Photonics). A standard curve using gallic acid (0–600 mg/mL) was used for quantification, and results were expressed as mg of gallic acid equivalents per 100 g of cheese (mg/100 g).

### Proteolysis index

To assess proteolysis, total nitrogen, water-soluble nitrogen at pH=4.6, and nitrogen soluble in 12% trichloroacetic acid were measured using the Kjeldahl method (*30*). Samples were prepared containing 10 g of cheese, and nitrogen fractions were quantified by analytical methods. Total protein content was calculated by multiplying the total nitrogen percentage by 6.38 (*30*). The proteolysis extension index (PEI)and the proteolysis depth index (PDI) were calculated (in %) using the following equations proposed by Pereira *et al.* (*33*, *34*):


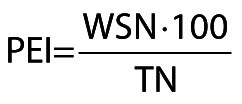
 /2/


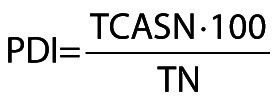
 /3/

where WSN is the content of water-soluble nitrogen (in %) at pH=4.6, TCASN is the content of trichloroacetic acid-soluble nitrogen (in %), and TN is total nitrogen (in %).

### Fatty acid profile

After lipid extraction according to Bligh and Dyer (*31*), a portion of the organic fraction containing 15 mg of lipids was converted into fatty acid methyl esters (FAMEs) following the method of Hartman and Lago (*35*), with modifications. The lipid fraction was mixed with 1 mL of 0.4 M methanolic potassium hydroxide solution (Êxodo Científica) and kept in a water bath (SL-150; Solab, Piracicaba, Brazil) at 100 °C for 10 min. The tubes were then cooled, and 3 mL of 1 M methanolic sulfuric acid solution (Êxodo Científica) were added, followed by incubation at 100 °C for another 10 min. After cooling, 2 mL of hexane (Sigma-Aldrich, Merck, St. Louis, MO, USA) were added, and the tubes were homogenized in a vortex (NA 3600; Norte Científica) for 10 s. The upper layer containing FAMEs dissolved in hexane was then collected for chromatographic analysis.

The fatty acid profile was analyzed using gas chromatography with a flame ionization detector (GC-FID 7820A; Agilent Technologies, Santa Clara, CA, USA). A volume of 1 µL of sample was injected in split mode (40:1), with the injector maintained at 240 °C. Hydrogen was used as the carrier gas at a constant flow rate of 1.5 mL/min. The FAMEs were separated on a DB-23 capillary column (60 m×0.25 mm×0.25 µm; Agilent Technologies) under a temperature program: an initial hold at 50 °C for 1 min, an increase to 175 °C at 25 °C/min, followed by a rise to 230 °C at 2 °C/min, with a final isothermal hold for 6 min. The detector temperature was set at 240 °C. FAMEs were identified by comparing retention times with those of the FAME Mix 37 standard (P/N 47885; Sigma-Aldrich, Merck). Quantification was carried out by normalization of peak areas, applying the flame ionization detector (FID) correction factors as described by Visentainer (*36*). Briefly, correction factors for each fatty acid were determined by analyzing known concentrations of the FAME Mix 37 standard, calculating the ratio between the theoretical and experimental relative responses. The corrected peak areas were then used to express the results as the percentage of the total chromatogram area, considering the ester-to-acid conversion.

### Color parameters

The color of the cheese paste and rind was evaluated by instrumental colorimetry according to the CIELAB system, with D65 illuminant, a 10º viewing angle, and calibration in specular component included (SCI) mode with a spectrophotometer (CM-5; Konica Minolta, Chiyoda, Japan). The Commission Internationale de L’Eclairage (CIE) color system was used, and the evaluated parameters were: L* value (luminosity), a* (+, red; -, green), b* (+, yellow; −, blue), chroma (C*), and hue angle (*h*°). From the primary color data, the total color difference (ΔE*), yellowness index (YI), browning index (BI) and color index (CI) were calculated according to the following equations, as described by Pathare *et al.* (*37*). For color analysis, the cheese was divided into three parts: the inner portion was used for interior color analysis, and the outer portion was used for rind color analysis:


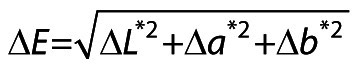
 /4/


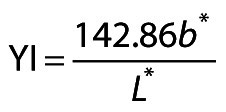
 /5/


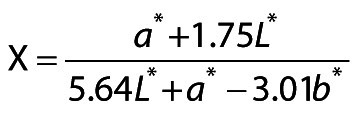
 /6/


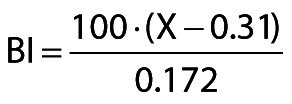
 /7/


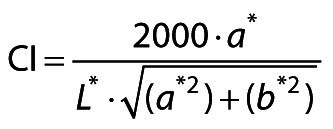
 /8/

#### Instrumental texture profile analysis

Textural properties were evaluated using a TA.XT Plus texture analyzer (Stable Micro Systems, Godalming, UK). Texture profile analysis (TPA) was performed with a 20 mm diameter cylindrical probe (P/20P) to assess hardness (N), adhesiveness (N·s), gumminess (N), chewiness (N), elasticity, cohesiveness and resilience. The operational parameters were: pre-test speed 1.0, test speed 0.5 and post-test speed 0.5 mm/s, compression distance 10.00 mm, trigger force 5×*g* (0.049 N), and load cell 50 kg. For the analysis, the cheese was divided into three parts, and the middle portion (approx. 100 g) was used for the texture test.

### Experimental design and statistical analysis

The experimental design was a completely randomized design (CRD) with four treatments (marination times) and three replicates. The data were analyzed using analysis of variance (ANOVA). When significant effects were identified, contrasts were performed to test the following hypotheses: (*i*) control *vs*. treatments, and (*ii*) bock beer *vs*. other marinades. The statistical analysis followed the experimental design and the multiple treatments. The assumptions of ANOVA were previously verified using the Shapiro–Wilk test to assess the normality of residuals and a test for homogeneity of variances, both at a 5% significance level. Statistical analyses, including ANOVA and contrast tests, were performed using R software, v. 4.4.3 (*38*), with a 5% significance level.

## RESULTS AND DISCUSSION

### Physicochemical evaluation of beer

Table 1 presents the results for the physicochemical parameters of the bock beer used for cheese marination.

**Table 1:** Characterization of the Bock beer used for cheese marination

Physicochemical property	
pH	4.38±0.02
TSPC as *γ(*GAE)/(mg/L)	478.0±7.3
L*	73.33±0.02
a*	13.54±0.03
b*	74.77±0.04
C*	75.98±0.04
*hº*	79.74±0.02
*γ*(melanoidin)/(g/L)	4.2±0.1

Results are expressed as mean value±standard deviation, *N*=3. TSPC=total soluble phenolic compounds, GAE=gallic acid equivalent, C*=chroma, *h*°=hue angle

The pH of the beer showed an acidic character, consistent with the typical values for this type of sample (*39*).

The high concentration of phenolic compounds (478 mg/L) and the melanoidin concentration observed in the analyzed bock beer can be attributed to the style of the beverage, which uses darker malts. The malting and roasting processes of barley promote the release of phenolic compounds and melanoidins (*23*). Thus, the combination of malt type and roasting process explains the higher concentrations of these compounds in this beer style, which is why bock beer was chosen for cheese marination.

According to Piazzon *et al*. (*40*), the total concentrations of polyphenols and phenolic acids vary considerably among different beer types, with values expressed as gallic acid equivalents, ranging from 366 μg/mL in alcohol-free beer to 875 μg/mL in Bock beers. In general, the highest concentrations were observed in bock, abbey, and ale beers, while the lowest were found in alcohol-free versions.

Zhao *et al*. (*41*) reported that the melanoidin concentration of beer ranged from 1.64 to 13.71 g/L. The variation in melanoidin concentration among different beers is due to differences in the raw materials used and the brewing process.

The instrumental color characterization of the beer suggests a predominance of intense yellow tones (high b* value) associated with a reddish contribution (positive a* value), resulting in a final golden-orange coloration. The high lightness (elevated L*) classifies it as a light-colored beverage, while the high chroma (C*) indicates a vivid tone. A predominance of the b* axis is observed, which is related to pigments derived from malt, as well as to products of Maillard and caramelization reactions occurring during thermal processing.

### Characterization of produced cheese

The mean values of physicochemical parameters, fatty acid profile, colorimetric parameters, and texture of the control cheese (C), T2, T4 and T6 (two, four and six days of marination, respectively) were determined, together with their respective standard deviations and the p-values from the analysis of variance (ANOVA). When the ANOVA p-value was significant (p<0.05), differences between sample pairs were evaluated using estimates of pairwise contrasts. No significant differences were observed for the fatty acid profile; therefore, no table of contrast estimates was generated for this variable.

The results indicated that the residuals could be considered normally distributed and that variances were homogeneous; therefore, data transformations were required only for variables with negative values, such as adhesiveness.

The analysis of variance revealed significant effects of the treatments on moisture, lipids, protein, ash, water activity (*a*_w_), and on the extent and depth of proteolysis (p<0.05) (Table 2). In contrast, carbohydrates, pH, and total phenolic compounds showed no significant differences (p>0.05).

**Table 2:** Composition and physicochemical characterization of cheese samples produced in the Diamantina region with and without bock beer marination

Physicochemical parameter	C	T2	T4	T6	p-value
	*w*(compound)/%				
Moisture	40.4±0.5	41.2±0.4	43.4±0.5	42.2±1.3	<0.001
Lipid	25.8±0.4	24.8±0.2	24.0±0.4	26.2±0.8	<0.001
Protein	25.6±0.1	27.00±0.06	26.4±0.4	26.1±0.1	<0.001
Ash	5.35±0.02	4.18±0.02	3.98±0.01	3.9±0.6	<0.001
Carbohydrate	2.9±0.9	2.820.6	2.2±0.7	1.6±0.8	0.222^ns^
*a*_w_	0.94±0.01	0.96±0.00	0.95±0.00	0.95±0.01	<0.001
pH	5.2±0.1	5.25±0.06	5.3±0.2	5.1±0.1	0.228^ns^
TSPC as *w*(GAE)/(mg/100 g)	138.7±3.5	134.1±3.2	143.7±6.5	145.6±5.6	0.0581^ns^
Proteolysis extension index/%	11.34±0.00	11.7±1.1	13.9±0.6	13.6±1.3	0.003
Proteolysis depth index/%	16.6±1.5	21.18±0.00	15.1±1.5	23.7±3.8	<0.001

Results are expressed as mean value±standard error, *N*=3, on a wet basis. *a*_w_=water activity, TSPC=total soluble phenolic compounds, GAE=gallic acid equivalent. ^ns^non-significant differences (p≥0.05). A p<0.05 indicates significant differences. C=control cheese (without marination), T2, T4 and T6=cheese marinated for 2, 4 and 6 days, respectively

Moisture mass fractions remained within the maximum limit of 45.9% (Table 2) established for Minas artisanal cheese (*25*). Contrast analysis (Table S1) indicated that T4 had a higher moisture content than the control (difference of 3.0 units; p<0.001) and T2 (difference of 2.19; p=0.002). Treatment T6 also showed higher values than the control (difference of 1.77; p=0.007). No significant differences were observed between C and T2 (p=0.225) or between T2 and T6 (p=0.134). A trend toward higher moisture value in T4 than in T6 was noted (p=0.050). These findings suggest that T4 and T6 contributed to increased moisture, with T4 being the most pronounced.

For lipid content, no differences were observed only between C and T6 (p=0.469) and between T2 and T4 (p=0.133) (Table S1). The results indicate that marination in beer significantly reduced lipid content at intermediate times (T2 and T4), an effect not maintained in T6, which showed values similar to the control (Table 2).

For protein, T2 showed higher values than the control (difference of 1.43 units; p<0.001), T4 (0.64; p=0.027) and T6 (0.94; p=0.003). Treatment T4 also resulted in higher values than the control (0.79; p=0.0083). No significant differences were found between C and T6 (p=0.084) or between T4 and T6 (p=0.386) (Table S1). These results demonstrate that all treatments increased protein content compared to the control, with T2 being the most effective (Table 2).

For total mineral content, all treatments differed significantly from the control (p<0.001), showing lower values: T2 (1.17; p<0.001), T4 (1.37; p<0.001) and T6 (1.46; p<0.0001) (Table S1). Among the treatments, T4 was lower than T2 (0.20; p<0.001), and T6 showed lower values than both T2 (0.29; p<0.0001) and T4 (0.08; p<0.001). These findings suggest that longer marination times reduce mineral content.

For water activity, all treatments showed a significant increase compared to the control: T2 (0.03; p<0.001), T4 (0.01; p=0.008) and T6 (0.02; p=0.002). Comparatively, T2 exhibited higher values than T4 (0.01; p=0.007) and T6 (0.01; p=0.032), with no differences between T4 and T6 (p=0.6845) (Table S1). This behavior indicates an initial increase in water activity at T2, followed by a reduction and subsequent stabilization (Table 2).

Beer marination significantly altered the cheese composition, reflecting both osmotic and biochemical effects. A similar pattern was observed for moisture and water activity: an initial increase during marination, followed by a reduction (Table 2). This may be associated with osmotic equilibrium established after four days of marination, when simultaneous water absorption and release by the cheese occur. This process was accompanied by reduced mineral mass fraction, suggesting possible migration of minerals from the cheese matrix into the marinating medium (*7*).

Lipid mass fraction showed a significant reduction at T2 and T4, suggesting possible extraction of liposoluble compounds by the alcoholic phase of the marinade. However, this effect was not sustained at T6, when values approached those of the control. In parallel, the increase in protein mass fraction, particularly at T2, may be associated with a relative concentration effect resulting from lipid and mineral losses, as well as selective retention of larger nitrogen fractions, while soluble fractions diffuse into the marinade (Table 2).

As this is an artisanal product, the cheese samples do not always have the same, but rather similar, mass. This variation could have influenced the absorption of the marinade and, consequently, the observed results.

Total carbohydrate mass fraction (Table 2), although it decreased slightly with marination time, did not show significant differences, suggesting that marination did not substantially affect the carbohydrate fraction of the cheese matrix. No significant changes were observed in pH either, indicating that bock beer marination did not markedly alter cheese acidity, possibly due to the buffering effect of the protein matrix.

Regarding total phenolic mass fraction, although no statistical significance was observed (Table 2), a trend of increasing values with marination time was noted. This may be related to the need for longer contact periods with the marinade to intensify the incorporation of phenolic compounds. This behavior is consistent with Cavalcanti *et al.* (*7*), who observed higher concentrations of TPC in goat coalho cheese after seven days of marination in red wine.

Proteolysis is responsible for changes in the maturation extension and depth indices, indicating the progress and intensity of the process. The extension quantifies the soluble high-molecular-mass peptides resulting from the proteolytic action of natural milk proteinases and rennet/coagulating enzymes on caseins, characterizing primary proteolysis. The depth is related to secondary proteolysis, which measures the formation of low-molecular-mass substances resulting from the enzymatic activity of the microbiota on the larger peptides generated in the early stages of maturation (*42*–*44*). The results obtained for the extension and depth of maturation indices are presented in Table 2.

The analysis of variance showed a significant effect of the treatments on the protein fraction soluble at pH=4.6 (extent of proteolysis; p=0.003) and on the fraction soluble in 12% TCA (depth of proteolysis; p<0.001). For the extent of proteolysis, T4 and T6 had higher values than the control (2.52; p=0.007 and 2.21; p=0.016, respectively). Among the treatments, T2 was lower than T4 (2.20; p=0.016) and T6 (1.89; p=0.035). No differences were found between C and T2 (p=0.934) or between T4 and T6 (p=0.936) (Table S1). These results indicate greater efficiency of T4 and T6 in enhancing primary proteolysis.

Regarding the depth of proteolysis, T2 and T6 showed higher values than the control (4.54; p=0.016 and 7.06; p=0.001, respectively). Among the treatments, T2 showed greater protein degradation than T4 (6.05; p=0.003), while T6 was higher than T4 (8.57; p<0.001). No differences were observed between C and T4 (p=0.564) or between T2 and T6 (p=0.194) (Table S1). These results demonstrate that T2 and T6 were the most effective in promoting a higher intensity of secondary proteolysis.

Since all the cheese samples were produced under the same conditions, using the same milk and ‘pingo’, the differences between the samples in the extension and depth indices can be attributed to the marination time, which significantly increased primary and secondary proteolysis. Immersion of the cheese in beer contributed to a higher relative humidity and consequently greater water activity (Table 2). The greater amount of free water in the cheese (*a*_w_) may have influenced the activity of protease enzymes. These findings corroborate those of de Oliveira Carneiro *et al.* (*43*), who found greater hydrolysis in cheeses during summer due to higher relative humidity, intensifying protease activity.

These results show that even at low temperatures ((12±1) °C), there was an acceleration in the maturation indices of the marinated cheese, particularly regarding the depth of maturation. This suggests that immersing the cheese in beer for six days accelerated secondary proteolysis, showing that this process could be an alternative to accelerate maturation and add value to the product due to the sensory characteristics triggered in the cheese by the hydrolysis of proteins.

Proteolysis plays a fundamental role in the sensory development of cheese, causing significant changes in texture, flavor formation, and the release of aromatic compounds throughout ripening. The hydrolysis of milk proteins releases peptides and free amino acids, which act as precursors of various compounds responsible for the characteristic aroma of cheese (*45*, *46*).

No significant difference (p≥0.05) was observed in the fatty acid composition (Table 3). In the control and all treatments, twenty-one distinct fatty acids were identified: thirteen saturated, four monounsaturated, and four polyunsaturated. The major fatty acids were palmitic acid (C16:0), with mass fractions ranging from 39.8% (T6) to 40.0% (T4), oleic acid (C18:1n9), ranging from 19.08% (T2) to 19.4% (C), and myristic acid (C14:0), ranging from 14.1% (C) to 14.2% (T6).

**Table 3:** Fatty acid profile of cheese samples produced in the Diamantina region with and without marination in bock beer

	Sample				p-value
C	T2	T4	T6
SFA	*w*(fatty acid)/%				
C4:0	1.33±0.09	1.36±0.04	1.37±0.02	1.5±0.1	0.143^ns^
C6:0	1.34±0.04	1.39±0.06	1.27±0.07	1.41±0.08	0.107^ns^
C8:0	1.11±0.01	1.16±0.05	1.17±0.06	1.08±0.03	0.136^ns^
C10:0	2.96±0.05	3.1±0.2	2.9±0.2	3.0±0.1	0.537^ns^
C11:0	0.06±0.00	0.06±0.00	0.06±0.01	0.06±0.00	0.998^ns^
C12:0	4.1±0.1	4.2±0.1	4.2±0.4	4.2±0.4	0.935^ns^
C13:0	0.14±0.01	0.14±0.00	0.15±0.01	0.14±0.01	0.520^ns^
C14:0	14.1±0.2	14.2±0.1	14.2±0.5	14.2±0.3	0.951^ns^
C15:0	1.37±0.00	1.37±0.01	1.37±0.02	1.37±0.02	0.933^ns^
C16:0	39.9±0.2	39.8±0.3	40.0±0.6	39.8±0.6	0.954^ns^
C17:0	0.68±0.02	0.69±0.04	0.70±0.02	0.68±0.02	0.815^ns^
C18:0	7.8±0.1	7.76±0.09	7.7±0.4	7.7±0.1	0.990^ns^
C20:0	0.15±0.01	0.14±0.01	0.14±0.01	0.16±0.01	0.142^ns^
∑SFA	75.03	75.37	75.13	75.35	-
MUFA					
C14:1	1.25±0.02	1.26±0.02	1.3±0.1	1.27±0.04	0.939^ns^
C16:1	1.61±0.02	1.62±0.01	1.63±0.05	1.60±0.01	0.604^ns^
C18:1n9t	0.36±0.01	0.35±0.00	0.36±0.02	0.35±0.00	0.530^ns^
C18:1n9c	19.4±0.2	19.08±0.08	19.3±0.4	19.2±0.3	0.592^ns^
∑MUFA	22.59	22.31	22.53	22.37	-
PUFA					
C18:2n6c	1.94±0.06	1.89±0.07	1.93±0.09	1.85±0.04	0.335^ns^
C18:3n3	0.21±0.01	0.20±0.00	0.20±0.00	0.20±0.00	0.559^ns^
C20:3n6	0.09±0.01	0.09±0.01	0.08±0.00	0.08±0.00	0.140^ns^
C20:4n6	0.14±0.00	0.14±0.00	0.13±0.01	0.14±0.00	0.295^ns^
∑PUFA	2.38	2.32	2.34	2.27	-

Results are expressed as mean value±standard error, *N*=3. C4:0=butyric acid, C6:0=caproic acid, C8:0=caprylic acid, C10:0=capric acid, C11:0=undecanoic acid, C12:0=lauric acid, C13:0=tridecanoic acid, C14:0=myristic acid, C15:0=pentadecanoic acid, C16:0=palmitic acid, C17:0=heptadecanoic acid, C18:0=stearic acid, C20:0=arachidic acid, C14:1=myristoleic acid, C16:1=palmitoleic acid, C18:1n9t=elaidic acid, C18:1n9c=oleic acid, C18:2n6c=linoleic acid, C18:3n3=alpha-linolenic acid, C20:3n6=dihomo-gamma-linolenic acid, C20:4n6=arachidonic acid, SFA=saturated fatty acids, MUFA=monounsaturated fatty acids, PUFA=polyunsaturated fatty acids. ^ns^non-significant differences (p≥0.05). A p<0.05 indicates significant differences. C=control cheese (without marination), T2, T4 and T6=cheese marinated for 2, 4 and 6 days, respectively

Margalho *et al.* (*47*) found a higher abundance of C16:0, followed by stearic acid (C18:0) and C18:1n9c in Canastra cheese, which were also predominant in Serro cheese. According to de Jesus Filho *et al*. (*48*), the composition of fatty acids is affected by the season and production units, as well as other factors, such as the animal diet. The feed provided to cattle in the summer consists predominantly of pasture, while in the winter, due to the drier climate, there is greater consumption of silage and feed, which directly impacts the amount and composition of specific fatty acids in milk.

The analysis of variance showed a significant effect of the treatments on the color parameters of both the rind and the interior of the cheese samples (p<0.05) (Table 4).

**Table 4:** Colorimetric parameters of cheese samples produced in the Diamantina region with and without marination in bock beer

Colorimetric parameter	C	T2	T4	T6	p-value
	Rind				
C*	17.8±1.1	36.9±3.5	32.6±5.0	29.2±0.9	<0.001
*hº*	84.2±077	69.7±1.1	71.2±1.4	71.2±1.2	<0.001
Δ*E*	-	28.4±3.6	23.1±8.0	19.7±2.7	-
YI	28.9±2.0	73.4±8.8	63.1±13.3	54.7±2.6	<0.001
DI	23.6±1.8	83.5±12.8	68.6±18.6	56.6±3.6	<0.001
CI	2.3±0.3	10.3±0.5	9.2±1.4	9.0±0.9	<0.001
	Cheese interior				
C*	18.2±1.4	19.8±1.2	18.4±2.7	17.0±1.1	<0.001
*hº*	84.2±0.6	83.6±0.6	84.3±0.7	83.9±0.7	0.005
Δ*E*	-	3.0±1.5	6.0±2.1	3.1±2.0	-
YI	28.8±2.8	32.1±2.4	29.8±5.4	26.7±2.0	<0.001
DI	23.4±2.7	26.7±2.4	24.4±5.	21.7±1.9	<0.001
CI	2.2±0.3	2.6±0.3	2.2±0.3	2.3±0.3	0.004

Results are expressed as mean value±standard error, *N*=3. *h°*=hue angle, C*=chroma, YI*=*yellowness index, DI=darkening index, CI=colour index. ^ns^non-significant differences (p≥0.05). A p<0.05 indicates significant differences. C=control cheese (without marination), T2, T4 and T6=cheese marinated for 2, 4 and 6 days, respectively

Regarding the rind, contrast analysis (Table S2) showed significant differences among all marinated cheese samples, as well as between the control and the treatments, for the chroma parameter (C*). The control cheese (C) exhibited significantly lower values than T2 (19.15; p<0.001), T4 (14.79; p<0.001) and T6 (11.47; p<0.001). Among the treatments, T2 showed higher values than T4 (4.37; p<0.001) and T6 (7.68; p<0.001), while T4 was higher than T6 (3.32; p=0.007). For the hue angle (*h*°), no significant differences were observed only between T4 and T6 (0.04; p=0.999). For the yellowness index (YI), the control cheese showed significantly lower values than T2 (44.47; p<0.001), T4 (34.21; p<0.001) and T6 (25.78; p<0.001). Among the treatments, T2 was higher than T4 (10.26; p<0.001) and T6 (18.69; p<0.001), and T4 exceeded T6 (8.44; p=0.008). Similarly, the browning index (BI) was significantly lower in the control cheese than in T2 (59.9; p<0.001), T4 (45.0; p<0.001) and T6 (33.0; p<0.001). Among the treatments, T2 had higher values than T4 (14.9; p<0.001) and T6 (26.9; p<0.001), while T4 exceeded T6 (12.0; p=0.008). For the color index (CI), no significant differences were observed only between T4 and T6 (0.26; p=0.783).

In the cheese interior, for chroma (C*), the control (C) showed significantly lower values than T2 (1.60; p=0.022). Among the treatments, T2 exhibited higher chroma than T6 (2.71; p<0.001), with no significant differences among the other treatments. For hue angle (*h*°), differences were observed between C and T2 (0.62; p=0.018) and between T2 and T4 (0.68; p=0.008). Regarding the yellowness index (YI), the control differed from T2 (3.36; p=0.014), while T2 showed higher values than T6 (5.41; p<0.001). Similar results were observed for the browning index (BI), with differences between C and T2 (3.24; p=0.011) and between T2 and T6 (5.02; p<0.001). Finally, the color index (CI) differed between C and T2 (0.31; p=0.007) and between T2 and T4 (0.30; p=0.010) (Table S2). Overall, the analysis of color parameters revealed distinct effects of bock beer marination on the rind and interior of Minas artisanal cheese.

On the rind, the changes were pronounced, indicating that marination promoted greater color intensity and browning. Chroma (C*) increased significantly in T2 compared to the control, reflecting higher color saturation and vividness, attributed to the incorporation of phenolic compounds and pigments from the beer. Hue angle (*h*°) decreased relative to the control, shifting the yellowish tone toward browner shades. This behavior was corroborated by increases in the yellowness index (YI) and browning index (BI), associated with the presence of melanoidins, polyphenols, and Maillard reaction products characteristic of bock beer (*23*). However, from T4 and T6 onward, a trend toward reduced C*, YI and BI values was observed, suggesting that the most pronounced effects of marination occur in the initial stages. Color perception decreased as marination time increased, as confirmed by Δ*E* determination. The longer the marination, the smaller the color difference of the cheese rind compared to the control.

In contrast, color changes in the cheese interior were minimal. C* and *h*° values remained stable across treatments, demonstrating preservation of the light yellow hue characteristic of Minas artisanal cheese. Likewise, YI, BI and CI showed no consistent differences, confirming the low penetration of beer compounds into the internal matrix, as confirmed by the total color difference (Δ*E*), which presented low values, indicating barely perceptible variations.

Overall, bock beer marination has a pronounced effect on the cheese surface, while the interior remains largely unchanged, preserving its original visual characteristics. This suggests that pigments present in the beer (melanoidins, furanoids and flavonoids) were primarily responsible for rind coloration, with greater absorption at the beginning of marination, followed by gradual degradation over time, likely due to enzymatic and/or oxidative reactions, a phenomenon also reported in studies of phenolic compound interactions with food matrices (*7*).

There was a significant difference (p<0.05) in the texture profile of the cheese samples, except for the resilience (p>0.05) (Table 5).

**Table 5:** Results of the instrumental texture profile analysis (TPA) of cheese samples produced in the Diamantina region with and without marination in bock beer

Texture parameter	C	T2	T4	T6	p-value
Hardness/N	56.6±6.2	32.8±3.0	60.4±17.5	44.1±0.5	<0.001
Adhesiveness/(N·s)	−4.0±1.4	−5.1±2.2	−9.7±5.6	−4.0±3.1	0.007
Cohesiveness	0.27±0.07	0.39±0.08	0.4±0.2	0.23±0.06	0.008
Gumminess/N	15.2±5.2	12.9±3.6	23.9±14.9	10.0±2.3	0.013
Chewiness/N	13.2±4.6	11.7±3.3	19.5±12.2	8.5±2.2	0.025
Springiness	0.86±0.05	0.91±0.03	0.82±0.07	0.85±0.05	0.030
Resilience	0.066±0.02	0.102±0.03	0.103±0.06	0.057±0.01	0.020

Results are expressed as mean value±standard error, *N*=3. A p<0.05 indicates significant differences. C=control cheese (without marination), T2, T4 and T6=cheese marinated for 2, 4 and 6 days, respectively

The analysis of variance revealed a significant effect of the treatments on hardness, adhesiveness, elasticity, cohesiveness, gumminess and chewiness (p<0.05) (Table 5).

In the contrasts for hardness, the control cheese (C) showed significantly higher values than T2 (23.82; p<0.001). Among the treatments, T4 showed greater hardness than T2 (27.63; p<0.001) and T6 (16.35; p=0.011), while no differences were observed between C and T4 (p=0.859), C and T6 (p=0.067), or T2 and T6 (p=0.115) (Table S3).

For adhesiveness, significant differences were observed between C and T4 (5.73; p=0.013) and between T4 and T6 (5.78; p=0.013). Regarding elasticity, a significant difference occurred only between T2 and T4 (0.09; p=0.019). For cohesiveness, T2 showed significantly different values from T6 (0.16; p=0.014). Concerning gumminess and chewiness, significant differences were detected exclusively between T4 and T6 (13.90; p=0.011 and 10.95; p=0.017, respectively) (Table S3). Finally, resilience did not differ significantly between the control and treatments, nor among the treatments themselves.

Cheeses T4 and C exhibited the highest hardness values, which, as shown in Table 2, were associated with higher crude protein content and lower percentages of secondary proteolysis. According to Barracosa *et al.* (*49*), proteolysis has a notable influence on cheese texture. Proteolysis releases amino and carboxyl groups, which significantly increase hydration and alter textural properties (*50*).

Marination for six days significantly reduced the cohesiveness, gumminess and chewiness of the marinated cheeses. This effect may be attributed to changes in the interactions between proteins and water that affect the cheese texture. During marination, compounds present in the marinade, such as acids and phenolic compounds, may interact with the protein matrix, altering its mechanical properties (*7*).

Cavalcanti *et al.* (*7*) observed that marinating goat coalho cheese in wine delayed secondary proteolysis. The marinated cheese showed greater hardness and chewiness but lower cohesiveness and elasticity than the control. These results suggest that in this case, the wine did not accelerate proteolysis but did influence the textural parameters. This could be explained by the inhibitory effect of wine compounds on proteolytic enzymes.

## CONCLUSIONS

Overall, beer marination affected the physicochemical and technological characteristics of the cheese in a time-dependent manner. Significant differences were observed in texture and proteolysis, with greater firmness in treatment T4, while T6 showed texture softening associated with the progression of proteolysis. Changes in moisture mass fraction, water activity, lipids, proteins and minerals were significant and reflected processes of absorption, diffusion and leaching into the marinade, while pH, carbohydrate mass fraction, and fatty acid composition were not significant and remained stable. Color changes, particularly in the rind, were significant. The cheese developed a brown–orange coloration due to the incorporation of pigments and phenolic compounds, suggesting a potential functional enhancement that remains hypothetical and requires confirmation through specific assays.

These findings refer to a study conducted at pilot scale, and variations in results may occur, particularly because the product is artisanal and subject to inherent variability in the production process. Nevertheless, beer marination represents a promising strategy to add value to artisanal Minas cheese. Future studies, including sensory evaluation and microbial activity analyses, are recommended to better understand product acceptance and microbiological behavior.

## SUPPLEMENTARY MATERIALS

Supplementary materials are available at: https://www.ftb.com.hr/images/pdfarticles/2026/April-June/FTB-64-256-S1.pdf.

## Appendix

**Table S1:** Estimates of pairwise contrasts (Tukey’s) for physicochemical variables

Variable	C–T2	C–T4	C–T6	T2–T4	T2–T6	T4–T6
Moisture	−0.81^ns^	−3.00 (p=0.000)	−1.77 (p=0.007)	−2.19 (p=0.002)	−0.96^ns^	1.23^ns^
Protein	−1.43 (p<0.001)	−0.79 (p<0.008)	-0.50^ns^	0.64 (p=0.026)	0.94 (p=0.003)	0.30^ns^
Lipid	0.99 (p=0.039)	1.72 (p=0.002)	−0.44^ns^	0.73^ns^	−1.43 (p=0.005)	−2.16 (p<0.001)
Ash	1.17 (p<0.001)	1.37 (p<0.001)	1.46 (p<0.001)	0.20 (p<0.001)	0.29 (p<0.001)	0.08 (p=0.001)
*a*_w_	−0.03 (p=0.000)	−0.01 (p=0.008)	−0.02 (p=0.002)	0.01 (p=0.007)	0.01 (p=0.032)	−0.00^ns^
Proteolysis depth index	−4.54 (p=0.016)	1.51^ns^	−7.06 (p=0.001)	6.05 (p=0.003)	−2.52^ns^	−8.57 (p<0.001)
Proteolysis extension index	−0.32^ns^	−2.52 (p=0.007)	−2.21 (p=0.016)	−2.20 (p=0.016)	−1.89 (p=0.035)	0.32^ns^

Results are expressed as estimates of pairwise contrasts (p-value), *N*=3. Estimates represent differences between treatment means. Negative values indicate higher means in the second treatment of the contrast. ^ns^non-significant differences (p≥0.05). p-values were adjusted using Tukey’s method. *a*_w_=water activity, TSPC=total soluble phenolic compounds, C=control cheese (without marination), T2, T4 and T6=cheese marinated for 2, 4 and 6 days, respectively

**Table S2:** Estimates of pairwise contrasts (Tukey’s) for rind and interior color parameters of the cheese

Variable	C–T2	C–T4	C–T6	T2–T4	T2–T6	T4–T6
	Rind					
*C**	−19.15 (p<0.001)	−14.79 (p<0.001)	−11.47 (p<0.001)	4.37 (p<0.001)	7.68 (p<0.001)	3.32 (p=0.007)
*h°*	14.54 (p<0.001)	13.03 (p<0.001)	13.07 (p<0.001)	−1.51 (p<0.001)	−1.47 (p<0.001)	0.04^ns^
CI	−7.97 (p<0.001)	−6.91 (p<0.001)	−6.65 (p<0.001)	1.05 (p=0.001)	1.31 (p<0.001)	0.26^ns^
DI	−59.90 (p<0.001)	−45.00 (p<0.001)	−33.00 (p<0.001)	14.90 (p=0.001)	26.90 (p<0.001)	12.00 (p=0.008)
YI	−44.47 (p<0.001)	−34.21 (p<0.001)	−25.78 (p<0.001)	10.26 (p=0.001)	18.69 (p<0.001)	8.44 (p=0.008)
	Cheese interior					
*C**	−1.60 (p=0.022)	−0.28^ns^	1.11^ns^	1.32^ns^	2.71 (p<0.001)	1.39^ns^
*h°*	0.62 (p=0.018)	−0.06^ns^	0.30^ns^	−0.68 (p=0.008)	−0.32^ns^	0.36^ns^
CI	−0.31 (p=0.007)	−0.01^ns^	−0.09^ns^	0.30 (p=0.010)	0.21^ns^	−0.08^ns^
DI	−3.24 (p=0.011)	−0.95^ns^	1.78^ns^	2.29^ns^	5.02 (p<0.001)	2.73 (p=0.042)
YI	−3.36 (p=0.014)	−0.99^ns^	2.05^ns^	2.37^ns^	5.41 (p<0.001)	3.04 (p=0.032)

Results expressed as estimates of pairwise contrasts (p-value), *N*=3. Estimates represent differences between treatment means. Negative values indicate higher means in the second treatment of the contrast. ^ns^non-significant differences (p≥0.05). p-values were adjusted using Tukey’s method. *h°*=hue angle, *C**=chroma, YI=yellowness index, DI=darkening index, CI=colour index, C=control cheese (without marination), T2, T4 and T6=cheese marinated for 2, 4 and 6 days, respectively

**Table S3:** Estimates of pairwise contrasts (Tukey’s) for texture profile parameters

Variable	C–T2	C–T4	C–T6	T2–T4	T2–T6	T4–T6
Hardness	23.820 (p=0.000)	−3.810^ns^	12.540^ns^	−27.630 (p<0.001)	−11.270^ns^	16.350 (p=0.011)
Adhesiveness	−1.108^ns^	−5.738 (p=0.013)	0.042^ns^	−4.630^ns^	1.150^ns^	5.780 (p=0.013)
Springiness	−0.048^ns^	0.038^ns^	0.010^ns^	0.085 (p=0.019)	0.058^ns^	−0.027^ns^
Cohesiveness	−0.120^ns^	−0.095^ns^	0.039^ns^	0.025^ns^	0.159 (p=0.014)	0.134 (p=0.047)
Gumminess	2.370^ns^	−8.630^ns^	5.270^ns^	−11.000^ns^	2.900^ns^	13.900 (p=0.011)
Chewiness	1.470^ns^	−6.300^ns^	4.650^ns^	−7.780^ns^	3.170^ns^	10.950 (p=0.017)
Resilience	−0.037^ns^	−0.037^ns^	0.009^ns^	−0.001^ns^	0.046^ns^	0.046^ns^

Results are expressed as estimates of pairwise contrasts (p-value), *N*=3. Estimates represent differences between treatment means. Negative values indicate higher means in the second treatment of the contrast. ^ns^non-significant differences (p≥0.05). p-values were adjusted using the Tukey’s method. C=control cheese (without marination), T2, T4 and T6=cheese marinated for 2, 4 and 6 days, respectively
